# Pathophysiology of Influenza D Virus Infection in Specific-Pathogen-Free Lambs with or without Prior *Mycoplasma ovipneumoniae* Exposure

**DOI:** 10.3390/v14071422

**Published:** 2022-06-28

**Authors:** Ema Robinson, Clyde Schulein, B. Tegner Jacobson, Kerri Jones, Jonathon Sago, Victor Huber, Mark Jutila, Diane Bimczok, Agnieszka Rynda-Apple

**Affiliations:** 1 Department of Microbiology and Cell Biology, Montana State University, 2155 Analysis Drive, Bozeman, MT 59718, USA; ema.f.robinson@gmail.com (E.R.); clyde.schulein@student.montana.edu (C.S.); bry.tegner@gmail.com (B.T.J.); krask@montana.edu (K.J.); mark.jutila@montana.edu (M.J.); diane.bimczok@montana.edu (D.B.); 2 Montana State Veterinary Diagnostic Laboratory, 1911 West Lincoln Street, Bozeman, MT 59718, USA; jonathon.sago@mt.gov; 3 Division of Basic Biomedical Sciences, Sanford School of Medicine, University of South Dakota, Vermillion, SD 57069, USA; victor.huber@usd.edu

**Keywords:** influenza D virus, *Mycoplasma ovipneumoniae*, polymicrobial infection, specific-pathogen-free sheep, respiratory coinfection, chronic nonprogressive pneumonia

## Abstract

Polymicrobial pneumonias occur frequently in cattle, swine, and sheep, resulting in major economic losses. Individual pathogens comprising these complex infections may be mild on their own but can instead exhibit synergism or increase host susceptibility. Two examples of such pathogens, *Mycoplasma ovipneumoniae* (*M. ovipneumoniae*) and influenza D viruses (IDVs), naturally infect domestic sheep. In sheep, the role of *M. ovipneumoniae* in chronic nonprogressive pneumonia is well-established, but the pathogenesis of IDV infection has not previously been studied. We utilized a specific-pathogen-free sheep flock to study the clinical response to IDV infection in naïve vs. *M. ovipneumoniae*-exposed lambs. Lambs were inoculated intranasally with *M. ovipneumoniae* or mock infection, followed after four weeks by infection with IDV. Pathogen shedding was tracked, and immunological responses were evaluated by measuring acute phase response and IDV-neutralizing antibody titers. While lamb health statuses remained subclinical, *M. ovipneumoniae*-exposed lambs had significantly elevated body temperatures during IDV infection compared to *M. ovipneumoniae*-naïve, IDV-infected lambs. Moreover, we found a positive correlation between prior *M. ovipneumoniae* burden, early-infection IDV shedding, and IDV-neutralizing antibody response. Our findings suggest that IDV infection may not induce clinical symptoms in domestic sheep, but previous *M. ovipneumoniae* exposure may promote mild IDV-associated inflammation.

## 1. Introduction

Polymicrobial pneumonias in cattle, swine, and sheep result in major annual economic losses in the livestock industry. It is hypothesized that during these complex infections, the primary pathogen may drive disease severity by remodeling or suppressing the host immune response, thereby facilitating infection by additional pathogens and/or limiting the host’s ability to combat the new infections [1,2,3,4,5,6]. Understanding the immunological processes driving these events is critical for effective prevention and management. Our group has investigated influenza-associated polymicrobial infections and identified several processes by which influenza A virus infection modulates host susceptibility to subsequent bacterial secondary infection [7,8,9]. We subsequently extended these investigations to include influenza D virus (IDV)-*Staphylococcus aureus* sequential infection using a mouse model [10]. In the current study we utilized a recently developed specific-pathogen-free (SPF) sheep flock to investigate the impact of prior *Mycoplasma ovipneumoniae* (*M. ovipneumoniae*) infection on IDV pathogenesis in a natural host [11].

*M. ovipneumoniae* is a respiratory bacterium commonly detected in healthy and diseased lambs [12,13,14]. *M. ovipneumoniae* infection can induce chronic nonprogressive pneumonia, particularly in 2- to 12-month-old lambs [15,16,17,18]. While its prevalence in healthy domestic lambs demonstrates that *M. ovipneumoniae* infection can be asymptomatic, evidence suggests that asymptomatic carriage may reduce lamb growth rates [19,20,21,22]. Importantly, while domestic sheep tolerate *M. ovipneumoniae* infection, wild sheep species such as Rocky Mountain Bighorn sheep (*Ovis canadensis*) and argali (*Ovis ammon*) are highly susceptible to *M. ovipneumoniae* infection, which predisposes them to severe-to-lethal polymicrobial interstitial pneumonia [19,20,21,23,24,25]. The mechanisms through which *M. ovipneumoniae* promotes secondary infection are not fully understood. Ex vivo studies have demonstrated that *M. ovipneumoniae* damages tracheal epithelium through the generation of reactive oxygen species, which may increase susceptibility to a secondary pathogen [4,26]. *M. ovipneumoniae* also induces ciliostasis [27], promotes anti-ciliary autoantibody production [28], and may compromise the mucociliary escalator and enable the migration of inert upper respiratory tract resident microbes such as *Mannheimia haemolytica* into the lower respiratory tract, which can result in severe interstitial pneumonia [23]. In the lower respiratory tract, *M. ovipneumoniae* reduces the phagocytic capacity of alveolar macrophages and impairs pathogen clearance [5,29], while stimulating proinflammatory cytokine secretion, which damages host tissue [4,30]. *M. ovipneumoniae* may also impair the adaptive immune response as it bears a surface protein that directly inhibits mitogen-stimulated expansion of T-cells and B-cells [31,32]. Each of these mechanisms may confer an immunosuppressed state and contribute to the increased disease severity associated with *M. ovipneumoniae* colonization.

IDVs comprise a recently identified genus of *Orthomyxoviridae* about which little is known [33,34]. The natural host range is broad, including (in order of known seroprevalence) livestock like cattle (40–95%) [35,36,37,38], swine (6–12%) [33,39,40], horses (12%) [41], sheep (2–6%) [38,39,42,43,44], goats (1–4%) [38,42,44,45], dromedary camels (6–99%, possible cross-reactivity with influenza C virus) [44,46], as well as wild ungulates including feral swine [40,47] and water buffalo [45]. There is also serological evidence of human infection [33,48,49,50], and experimental infections have been carried out in model organisms of the human respiratory tract (mice [10,51], guinea pigs [52], and ferrets [33,53]). In most species, IDV primarily infects ciliated cells of the upper respiratory tract and soft palate [4,33,53,54]. Cellular tropism varies between species due in part to differences in respiratory system glycosylation patterns [54,55]. IDV infections depend on docking of the major surface glycoprotein (hemagglutinin-esterase fusion protein) to extracellular 9-*O*-acetylated sialic acid residues, and as such, cellular permissivity is dependent on the presence of these modified glycans [56,57]. IDV infections typically remain subclinical [35,53,58,59,60,61], but metagenomics studies suggest that IDVs may play an etiologic role in a bovine respiratory disease (BRD), a severe-to-lethal polymicrobial infection common in pre- and post-weaned calves [35,62,63]. In cattle, IDVs infect both the upper and lower respiratory tract and can cause mild-to-moderate respiratory symptoms [53,58,61,64]; however, respiratory tissue tropism has not been established for most hosts, including sheep. Until now, active IDV infection has never been documented in sheep, but serostudies in the United States [42], Ireland [43], France [38], and West Africa [39,44] report IDV-neutralizing antibodies in domestic sheep, suggesting that IDV infections in sheep occur commonly across multiple continents.

Despite evidence of circulation of both *M. ovipneumoniae* and IDV in sheep, no studies have characterized infections involving both pathogens. Our previous work and the work of others suggests that *M. ovipneumoniae* monoinfection does not consistently induce clinical illness in SPF lambs [11,19,65,66]. To date, experimental coinfection of an IDV with a bacterial pathogen has been limited to two studies in calves and one in mice [10,58,64]. In the current study, we infected SPF lambs with *M. ovipneumoniae* or mock treatment. After four weeks, we infected all animals with IDV to characterize IDV pathogenesis with and without a recent or ongoing *M. ovipneumoniae* infection. We found that on their own, both *M. ovipneumoniae* and IDV infections were asymptomatic. While we observed no significant difference in overt respiratory disease or shedding when *M. ovipneumoniae*-infected versus naïve lambs were inoculated with IDV, we found subclinical (<40 °C) elevation in rectal temperature, as well as elevated serum protein concentrations in lambs exposed to both pathogens. Our data also allowed for correlative analysis which suggests that the amount of recent *M. ovipneumoniae* burden may correspond to the degree of early-phase IDV replication and the endpoint IDV-neutralizing antibody titer. Because no causal relationship has been established, these data must be confirmed by additional experiments.

## 2. Materials and Methods

### 2.1. Animals and Husbandry

All procedures in this study were approved by the Institutional Animal Care and Use Committee (IACUC) of Montana State University, protocol# 2019-109. We previously reported the derivation of a SPF sheep flock through established motherless rearing methods using mixed-breed (Rambouillet/Targhee) founders (F0) [11]. The SPF flock was maintained under SPF conditions at the Johnson Family Livestock Facility (JFLF) in Bozeman, MT. Of the F1 SPF sheep, 2 rams and 11 ewes were bred, and of the resulting progeny (F2), 3 ewes and 4 wethers were selected for each experimental group in this study (*n* = 7 lambs/group).

Four days prior to the study start date, 12–15-week-old lambs were weaned and moved into a temperature-controlled room (15.5–16.8 °C) in the JFLF ABSL-2 facility for acclimation. Experimental groups were housed in different rooms, separated by a procedure area. To minimize the possibility of cross-contact or accidental exposures, all personnel showered and donned sterile personal protective equipment before and after entering either room. All protocols were performed on the *M. ovipneumoniae*-naïve group prior to the *M. ovipneumoniae*-inoculated group. Equipment was sterilized between experimental groups and, when possible, separate sets of procedural equipment were used for each group.

### 2.2. Health Monitoring

Lamb health status was monitored daily, and combined clinical scores were assigned daily using previously established metrics [11]. To summarize, lambs were scored in five categories: (1) behavior, (2) appetite, (3) respiratory symptoms, (4) clinical interventions, and (5) body temperature (measured rectally). Each category uses a scale from 0 to 5 with higher scores indicating a worsening state, giving a maximum possible combined clinical score of 25. Lambs were also weighed every 14 days. Trained animal husbandry staff examined lambs twice daily and monitored them by video feed throughout each day.

### 2.3. M. ovipneumoniae Preparation

*M. ovipneumoniae* inoculum was grown from a previously described nasal wash isolate (MSU NW-4) and expanded exactly as described in the original publication [11]. Briefly, inoculum was grown at 37 °C in Mycoplasma broth under microaerophilic conditions. On the day of inoculation, media was removed (10,000× *g*, 10 min, 4 °C) and culture was resuspended in sterile FACS buffer (2% fetal bovine serum and 0.1% sodium azide in DPBS) for counting by flow cytometry [67]. Cells were subsequently stained with SYBR Safe DNA Gel Stain (Invitrogen, Carlsbad, CA, USA) at 4 °C for 30 min and Absolute Counting Beads (Invitrogen, Carlsbad, CA, USA) were added to solution immediately prior to analysis using an Accuri C6 flow cytometer (BD Biosciences, Franklin Lakes, NJ, USA).

### 2.4. Virus Preparation

Influenza D virus (D/swine/Oklahoma/1334/2011) was propagated in embryonated chicken eggs, and inoculum titer was determined by plaque assay as previously described with modifications [68,69]. On the day of inoculation, an aliquot of inoculum was serially diluted in 1X inoculation media (1X MEM, 0.3% bovine serum albumin, 2 mM HEPES, with penicillin/streptomycin) containing 1 µg/mL TPCK-trypsin (Thermo Fisher Scientific, Waltham, MA, USA). Dilutions were plated in duplicate onto 90–100% confluent monolayers of Madin-Darby Canine Kidney cells (MDCK, ATCC# NBL-2), a kind gift from Dr. Benfeng Lei. After a 1 h infection period at 33 °C, cells were washed, and a semisolid overlay was added (1X inoculation media, 1 µg/mL TPCK-trypsin (Thermo Fisher Scientific, Waltham, MA, USA), 0.6% agarose, 100 µg/mL DEAE-dextran (Sigma Aldrich, St. Louis, MO, USA)). Plates incubated for 5 days at 33 °C. Plaques were visualized by tetrazolium dye vital staining by dispensing 0.5 mL dye solution (3 mg/mL 3-(4,5-Dimethylthiazol-2-yl)-2,5-Diphenyltetrazolium Bromide (MTT, Thermo Fisher Scientific, Waltham, MA, USA) in 150 mM NaCl) on top of each overlay as previously described [70]. Cells were incubated with the dye for 60 min at 37 °C, 5% CO_2_, or until blue puncta developed and plaques were enumerated immediately.

IDV for hemagglutinin inhibition assays was propagated in swine testicular cells (ST, ATCC# CRL-1746, a kind gift from Dr. Feng Li). Upon reaching 60% confluence, cells were inoculated (MOI = 0.1) in a minimal volume of inoculation media at 33 °C, 5% CO_2_ for 1 h. Cells were then cultured for 5 days at 37 °C, 5% CO_2_ in inoculation media with TPCK-trypsin reduced to 0.1 µg/mL. After outgrowth period, media was collected and virus was concentrated by ultracentrifugation over a 30% sucrose cushion at 100,000× *g*, 90 min, 4 °C. Pellets were resuspended in sterile DPBS and stored at −80 °C until use.

### 2.5. Intranasal Inoculations

Each experimental group consisted of 3 ewes and 4 wethers aged 12–15 weeks old. Among the entire cohort were 3 sets of twins, which were distributed evenly across groups. On the first day of the study, lambs were inoculated with either sterile DPBS (*n* = 7) or 3.5 × 10^8^ CFU *M. ovipneumoniae* (*n* = 7) by instilling 15 mL into each naris and 10 mL orally similar to what has previously been described [11,25,71,72,73]. On day 28 of the study, when in our previous study the peak *M. ovipneumoniae* infection occurred in SPF sheep [11], all lambs were inoculated intranasally with 1.86 × 10^5^ PFU egg grown IDV in DPBS as previously described, with modifications [33,74].

### 2.6. Sampling

#### 2.6.1. Nasal and Rectal Swabs

At indicated timepoints, either both nares or rectum were swabbed, and swabs were transported in DPBS (2 mL). Swabs were vortexed briefly and transport media was aliquoted and stored at −20 °C until use.

#### 2.6.2. Serum Collection

At the stated timepoints, whole blood was collected by jugular venipuncture using BD Vacutainer serum tubes (Becton, Dickson and Company, Franklin Lakes, NJ, USA) and serum was separated by centrifugation at 1000× *g*, 10 min, 4 °C. Aliquots were stored at −20 °C until use.

#### 2.6.3. Fecal Samples

Fecal samples were obtained directly from the rectum and were transported on ice. Samples (80–120 mg) were subsequently homogenized in 500 µL TRIzol^TM^ Reagent (Invitrogen, Carlsbad, CA, USA) using a bead beater, then stored at −80 °C until time of RNA extraction.

### 2.7. Determining Pathogen Burden

#### 2.7.1. *M. ovipneumoniae* Quantification

DNA was extracted from nasal swab samples (500 µL) using the DNeasy Ultraclean Microbial Kit (Qiagen, Redwood City, CA, USA), with modifications. Briefly, cells were pelleted at 10,000× *g* for 10 min, resuspended in lysis buffer, and incubated at 95 °C for 10 min prior to proceeding with the remainder of the manufacturer’s protocol. Each sample was eluted in 50 µL of provided elution buffer. *M. ovipneumoniae* burden was determined by qPCR using SsoAdvanced Universal SYBR Green Supermix (Bio-Rad Laboratories, Hercules, CA, USA) and the following previously validated primers [75]: Fwd: 5′-TCTCCCAGATGATGCTAACC-3′; Rev: 5′-TGAAAATCAACTGGTCTAA-3′. Samples were volume-normalized using 0.5 µL DNA per reaction. Genome copy number was interpolated from a standard curve using a fragment of the *p113* gene (Twist Bioscience, San Francisco, CA, USA; sequence listed below). A standard curve was analyzed in each sample plate. Thermal cycling reactions proceeded in 10 µL volumes using a CFX384 Touch Real-Time PCR Detection System (Bio-Rad Laboratories, Hercules, CA, USA) with the following cycling conditions: 3-min polymerase activation at 98 °C followed by 40 cycles of denaturation for 15 s at 98 °C and annealing/extension for 30 s at 60 °C.

*p113* fragment sequence (5′->3′): AGCTACCAAGTCGACATTCTAGAAATTCTCCCAGATGATGCTAACCAAAATTTT AAAGTCAAATTTCAAGCTAGCCAAAAATTAGCAAACGGTGACATCGCCAAATCT GACATTTATGAACAAGTTGTTTCTTTTGTCAAAGAATCAACTATTTTAATTGCCGA ATTTAATTTTTCCTTACAAAAAATTACAAGCAGACTTAATCAACAAGTCCAAAAT TTAATTTCTGCTCGAACCGCCAATTTTGCTGATCAAAATTCAGCTACTTCAAATC CAACAGATCCTAGCACAATTAGACCAGTTGATTTTCAACATGACTTAAGAATTC ATAAAGCAAA

#### 2.7.2. IDV Quantification

Viral RNA was extracted from nasal and rectal swabs (140 µL) with the QIAamp Viral RNA Mini Kit (Qiagen, Redwood City, CA, USA) as directed, using the provided carrier RNA, and eluted in 60 µL nuclease-free water. Fecal samples were processed exactly as previously described [76]. Blood samples (100 µL) were processed using the PureLink™ RNA Mini Kit (Invitrogen, Carlsbad, CA, USA) according to the manufacturer’s instructions. All RNA was reverse-transcribed using High-Capacity cDNA Reverse Transcriptase Kit (Applied BiosciencesTM, Cheshire, UK) using 10 µL RNA extract per reaction. Prior to IDV quantification, successful RNA extraction was confirmed using qRT-PCR to probe for pan-eukarya 18S rRNA (Hs99999901_s1; Applied Bioscience, Cheshire, UK). Extractions with Cq > 25 were excluded from analysis and extraction was repeated. IDV burden was determined by qRT-PCR using SsoAdvanced Universal Probes Supermix (Bio-Rad Laboratories, Hercules, CA, USA) and the following previously validated FAM-labeled primers targeting the segment encoding PB1 [76]: Fwd: 5′-CAGCTGCGATGTCTGTCATAAG-3′; Rev: 5′-ACAAATTCGCAGGGCCATTA-3′; Probe: 5′-FAM-AATGGACTTTCTCCTGGGACTGCT-TAMRA-3′. To generate a standard curve for interpolation of PFU equivalents (PFU Equiv.), IDV inoculum was serially diluted in sterile DPBS and RNA was extracted from each dilution as described for nasal and rectal swabs. A plaque assay was performed in parallel as described above to confirm the inoculum concentration. cDNA libraries were generated as described above and the resulting standard curve was analyzed in each sample plate. Thermal cycling reactions proceeded as for *M. ovipneumoniae* except with the following cycling conditions: 30 s polymerase activation at 95 °C followed by 40 cycles of denaturation for 15 s at 95 °C and annealing/extension for 30 s at 60 °C.

### 2.8. Hemagglutinin Inhibition (HI) Assay

IDV neutralizing antibody titers were determined by HI as previously described, with minor modifications [77]. Briefly, serum was treated with receptor-destroying enzyme II (Denka Seiken, Tokyo, Japan) at a 3:1 ratio for 18 h at 37 °C, followed by enzyme inactivation at 56 °C for 30 min. Sera were diluted to 0.1X in PBS and stored at −20 °C until use. HI was performed on sera diluted in series from 1:10 to 1:1280 using a 1% suspension of washed chicken red blood cells (Lampire Biological Laboratories, Pipersville, PA, USA) and in the presence of 4 HAUs of ST-grown IDV. The agglutination reaction proceeded for 45 min at room temperature in V-bottom plates and titers were read by tilting the plate 90° for 30 s. Viral HA activity was determined just prior to test and confirmed with an in-plate dilution series. Tests were performed on two independent days, twice each day. Partially agglutinated wells were considered agglutinated. Titers are reported as inverse dilution, and sera with titers < 10 were assigned a titer of 5 for statistical analysis and graphical representation.

### 2.9. Acute Phase Protein and L-Lactate Quantification

Total protein was measured by BCA assay as directed (Thermo Fisher Scientific, Waltham, MA, USA). Albumin was determined using a modified bromocresol green (BCG) protocol. Briefly, 50 µL BCG reagent (Eagle Diagnostics, De Soto, TX, USA) was combined with 50 µL serum diluted appropriately in 0.9% saline. Reaction incubated 15 min at room temperature before measuring absorbance at 630 nm. Each test plate included a 125–1500 µg/mL standard curve using a commercially-available 2 mg/mL bovine serum albumin standard (Thermo Fisher Scientific, Waltham, MA, USA). Serum amyloid A was measured using a commercially available ELISA (PHASE™ RANGE Multispecies SAA ELISA kit, Tridelta Development, Ltd., Maynooth, Co., Kildare, Ireland) using the bovine standard. L-lactate was measured using the EnzyChrom™ L-lactate Assay Kit (BioAssay Systems, Hayward, CA, USA) as directed, scaled to a final volume of 100 µL/reaction. Sera exhibiting gross hemolysis (background-corrected absorbance at 520 nm > 0.5) were omitted from statistical analyses.

### 2.10. Statistical Analyses

Data are presented as means of at least two technical replicates per sample. Unless otherwise stated in figure legend, differences between treatment groups were analyzed using unpaired two-tailed Student’s t-test and all data are reported as means ± standard deviation (SD). When multiple comparisons were made, *p*-values were adjusted by the Bonferroni–Dunn method. Confidence in Spearman’s rank-order correlation analyses was expressed using a two-tailed *p*-value. All analyses were performed using GraphPad Prism 9 software.

## 3. Results

### 3.1. Prior M. ovipneumoniae Infection Elicits a Subclinical Response in IDV-Infected Lambs

**Figure 1:**
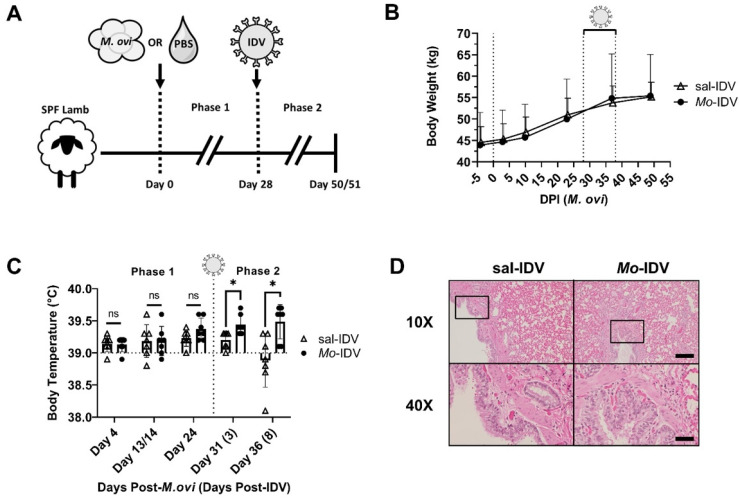
IDV with or without recent *M. ovipneumoniae* infection does not induce clinical symptoms in SPF lambs. (**A**) Schematic representation of experimental design. On day 0, seven lambs were administered *M. ovipneumoniae* (3.5 × 10^8^ CFU) or PBS control (*n* = 7 lambs/group); at day 28, all lambs were inoculated with IDV (D/Oklahoma/1337/2011; 1.86 × 10^5^ PFU). (**B**) Weights and (**C**) rectal temperatures were monitored throughout the study. (**D**) Representative histological analysis of distal lungs from each treatment group. Boxes on 10× images denote 40× field. 10× scale bar: 500 μm; 40× scale bar: 100 μm; *M. ovi*: *M. ovipneumoniae*; comparisons evaluated by Student’s *t*-test; * Bonferroni–Dunn-adjusted *p* < 0.05, ns: *p* > 0.05. Vertical dashed lines denote IDV shedding period (**B**) or inoculation (**C**), horizontal line indicates average clinically normal lamb rectal temperature (39 °C). Data are represented as mean ± standard deviation (SD).

To assess IDV pathogenesis with and without prior *M. ovipneumoniae* infection, fourteen SPF lambs were divided into two groups housed in separate rooms (*n* = 7 lambs/group). On day 0 (initiation of Phase 1), *M. ovipneumoniae* (3.5 × 10^8^ CFU) or phosphate-buffered saline sham were introduced intranasally. After 4 weeks, all lambs were intranasally inoculated with IDV (1.86 × 10^5^ PFU), resulting in a double-infected group (*Mo*-IDV) and an *M. ovipneumoniae*-naïve group infected only with IDV (sal-IDV) (Figure 1A). Lambs were monitored for an additional 22 (sal-IDV) or 23 (*Mo*-IDV) days before necropsy. For clarity, the first four weeks of the study during which time we evaluated the difference between *M. ovipneumoniae*-inoculated lambs and immunologically naïve saline controls is denoted as phase 1, and the time period after IDV infection, comparing IDV pathogenesis with versus without prior *M. ovipneumoniae* exposure, is denoted as phase 2 (Figure 1A).

Overall health, respiratory symptoms, demeanor, weight, and rectal temperature were documented throughout the study to identify signs of illness (Table 1 and Figure 1B,C). For reasons deemed unrelated, the experimental conditions, one sal-IDV wether (4104) was euthanized 13 days post-IDV after exhibiting signs of abdominal discomfort and anorexia starting 8 days post-IDV. Data collected from this lamb after 7 days post-IDV were omitted from analysis.

As expected, based on our previous work, during phase 1 of the study, *M. ovipneumoniae*-infected and sham-treated lambs remained asymptomatic, gained weight at the same rate, and showed no significant difference in rectal temperatures (Table 1 and Figure 1B,C) [11]. Following IDV infection (phase 2), we observed no clinical manifestations in any lamb, and no difference in growth between groups (Table 1 and Figure 1B). We did find that *Mo*-IDV lambs had significantly higher rectal temperatures versus sal-IDV lambs at both 3 and 8 days post-IDV (Table 1 and Figure 1C). While all temperatures remained in the normal range (<40 °C) [78], this finding could indicate that recent *M. ovipneumoniae* infection promotes a mild inflammatory response during subsequent IDV infection.

Necropsy was performed on 5–6 lambs per group at 22 (sal-IDV) or 23 (*Mo*-IDV) days post-IDV. All lambs in both groups were apparently healthy at necropsy, with no abnormalities or signs of pathogen-associated inflammation. Lung tissue featured mild-to-moderate congestion, petechiae, hemorrhage, edema, and/or atelectasis, all of which were deemed agonal by a board-certified veterinary pathologist. Accordingly, histological evaluation showed no evidence of microscopic lesions, and no evidence of inflammation-associated immune cell recruitment (Figure 1D). Similarly, concurrent evaluation of bronchoalveolar lavages collected at necropsy did not reveal any differences in either numbers or composition of cellular infiltrates between sal-IDV and *Mo*-IDV infected lambs (data not shown).

### 3.2. Recent M. ovipneumoniae Infection Has a Minimal Impact on IDV Shedding

**Figure 2:**
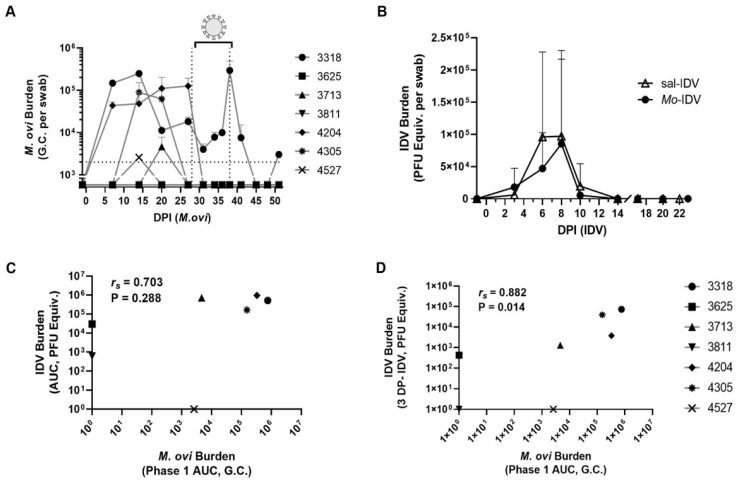
Recent *M. ovipneumoniae* infection correlates to early IDV shedding. Nasal shedding was monitored over time for (**A**) *M. ovipneumoniae*, individual lambs and (**B**) IDV, mean for each group. (**C**,**D**) Scatterplot showing correlation between total *M. ovipneumoniae* burden and (**C**) total IDV shedding, and (**D**) IDV shedding at 3 days post-IDV. Dotted horizontal line represents assay detection limit. Phase 1 AUC: area under the curve for phase 1 section of traces in (**A**); G.C.: enumeration of genome copies by qPCR; PFU Equiv.: enumeration of plaque-forming units by qRT-PCR; *r_s_*: Spearman correlation coefficient; P: *p*-value. Data presented as mean ± SD.

Nasal swabs were collected throughout the study to determine bacterial and/or viral burden. Pathogen burden (*M. ovipneumoniae*: Genome Copies, G.C.; IDV: plaque-forming unit equivalents, PFU Equiv., see methods for details) was quantified by qPCR or RT-qPCR using the standard curve method (Figure 2A,B and Supplementary Figure S1A,B). We found that after intranasal inoculation, nasal *M. ovipneumoniae* shedding varied widely between lambs and over time (Figure 2A). Shedding was never detected for two wethers (3625 and 2811). One ewe and one wether (4527 and 3713) were *M. ovipneumoniae*-positive at only one timepoint (day 14 or 20, respectively), and one wether (4305) shed only at the day 14 and day 20 timepoints. Two ewes (3318 and 4205) shed *M. ovipneumoniae* throughout phase 1 (until 1 day prior to IDV inoculation) and were presumably shedding at the time of IDV infection. Of those two, *M. ovipneumoniae* titers dropped sharply at the next collection timepoint (3 days post-IDV) and remained undetectable for the duration of the study (4205) or remained relatively low (3318) until IDV shedding resolved (Figure 2B and Supplementary Figure S1A). Presence of IDV in blood (at day 3 post-IDV) and rectal swabs (at day 7 post-IDV) was determined based on the pattern of nasal IDV shedding in Figure 2B. At these selected time points, we found no signs of viremia or rectal shedding (data not shown).

All lambs were inoculated intranasally with IDV at the start of phase 2 (28 days post-*M. ovipneumoniae*). We found no significant difference in IDV shedding between groups at any discrete collection timepoint and no difference in total shedding as reflected by area under the curve (AUC) (Figure 2B and Supplementary Figure S1A,B). IDV remained undetectable for one ewe from each group (sal-IDV: 4016, *Mo*-IDV: 4527) (Supplementary Figure S1A,B). The remaining 6 sal-IDV lambs and 5 of the remaining 6 *Mo*-IDV lambs shed IDV at 3, 6, 8, and 10 days post-IDV. One *Mo*-IDV ewe (3811) shed only at 6, 8, and 10 days post-IDV. While not significant, *Mo*-IDV lambs showed a trend towards decreased IDV burden compared to sal-IDV animals at 6 days post-IDV (sal-IDV = 9.60 × 10^4^ ± 1.32 × 10^5^ PFU Equiv.; *Mo*-IDV = 4.71 × 10^4^ ± 5.55 × 10^4^ PFU Equiv.; ratio-paired *t*-test, adjusted *p*-value = 0.08, Figure 2B).

The wide distribution in degree and timing of shedding of both pathogens allowed us to assess whether the amount of *M. ovipneumoniae* shedding correlated to the amount of IDV shedding within the *Mo*-IDV lambs. To test this, we utilized a Spearman’s rank-order correlation analysis, which separately ranked lambs by order of their degree of IDV shedding and by order of their *M. ovipneumoniae* shedding, then determined how similar those rankings are between the two rank orders. This test describes both the strength and directionality of the correlation (Spearman’s coefficient, *r_s_*; positive correlation: 0 to 1, negative correlation: 0 to −1, with 1 or −1 being perfect correlations) as well as the certainty of that strength (a standard *p*-value, P). We found a strong positive correlation between total *M. ovipneumoniae* shedding during phase 1 of the study (days 0–28 post-*M. ovipneumoniae*) and total IDV shedding, but this correlation had weak certainty (*r_s_* = 0.703, *p* = 0.228) (Figure 2C). Interestingly, we found a very strong positive correlation with high certainty between phase 1 *M. ovipneumoniae* shedding and subsequent IDV shedding at 3 days post-IDV (*r_s_* = 0.882, *p* = 0.014) (Figure 2D). While causation cannot be determined, these data suggest that *M. ovipneumoniae* infection is associated with subsequent accelerated early-infection IDV replication.

### 3.3. Total Serum Protein and Acute Phase Response

**Figure 3:**
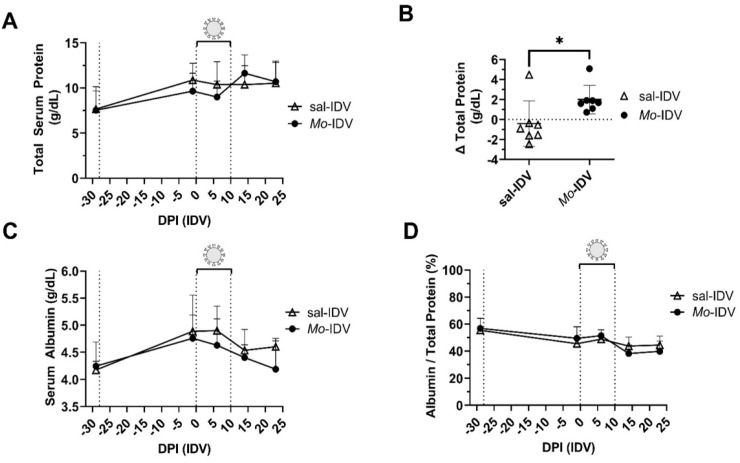
Prior infection with *M. ovipneumoniae* has a minimal effect on IDV-associated acute-phase response. (**A**) Total serum protein was measured throughout duration of study. (**B**) Change in total serum protein between 1 day prior to IDV infection and 14 days post-infection. (**C**) Serum albumin concentration were measured throughout duration of study. (**D**) Ratio of serum albumin:total protein. Vertical dotted lines signify inoculation with *M. ovipneumoniae* or IDV, or the last observed timepoint of IDV shedding, respectively. DPI: days post-infection. Data are presented as mean ± SD. Comparisons were evaluated by Student’s *t*-test. * Bonferroni–Dunn adjusted *p* < 0.05.

Increased total protein in serum can occur during prolonged infection due to the generation of acute-phase proteins during an inflammatory response. We collected serum every 7 or 14 days throughout the study and found no difference in total serum protein levels between treatment groups at any serum collection timepoint (Table 2 and Figure 3A). Because the total serum protein response occurs over time and may not be reflected by single-timepoint or even sequential-timepoint comparisons, we also compared total serum protein concentrations from serum collected before versus after the IDV shedding period (1 day prior to IDV and 14 days post-IDV) (Figure 1B). Consistent with our earlier findings that recent *M. ovipneumoniae* infection may increase IDV-associated body temperature and early viral replication, we found that *Mo*-IDV lambs also showed an increase in total serum protein concentration after IDV infection, while sal-IDV lambs showed a small decrease in total serum protein during this time period (*Mo*-IDV: 2.00 ± 1.43 g/dL versus sal-IDV: −0.413 ± 2.27 g/dL; adjusted *p*-value = 0.035) (Figure 3B).

We also measured acute-phase response via serum albumin (a negative acute-phase protein), serum amyloid A (a positive acute-phase protein), and L-lactate (a metabolic marker of critical illness). As expected, each group displayed decreased serum albumin levels following IDV infection (14 days post-IDV), although this change was not statistically significant (Table 2 and Figure 3C). Albumin fraction (albumin/total protein) remained largely stable, and no significant differences were observed between groups at any timepoint, indicating overall relative health (Figure 3D). Serum amyloid A levels varied over time and between lambs but were not affected by treatment (Table 2).

While it has been used as a prognostic indicator in some species, the predictive power of L-lactate in sheep infection outcomes remains unexplored in a controlled infection setting. To investigate whether L-lactate was a predictor of IDV-associated pathology, we compared serum L-lactate at 1 day prior to and 6 days post-IDV and found no significant difference between experimental groups at either timepoint (Table 2). L-lactate also failed to predict viral load or reflect past (phase 1) or ongoing bacterial burden (correlative data not shown).

### 3.4. IDV Infection Elicits a Neutralizing Antibody Response in SPF Lambs

**Figure 4:**
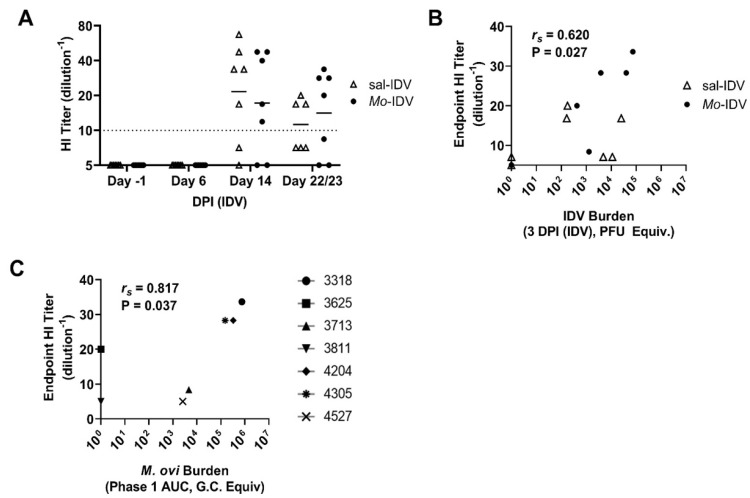
IDV antibody response correlates to prior *M. ovipneumoniae* burden and early-IDV-infection viral load. (**A**) Neutralizing antibody titer by HI assay. (**B**,**C**) Correlations between HI titer and (**B**) viral burden at 3 days post-IDV or (**C**) total *M. ovipneumoniae* burden during phase 1 (Phase 1 AUC). AUC: area under the curve; *r_s_*: Spearman correlation coefficient; P: *p*-value. Horizontal line denotes HI cutoff value (10).

To date, studies have reported domestic sheep seroconversion against D/OK, D/660, and D/IM lineage IDVs [42]. To investigate the induction of a neutralizing antibody response in sal-IDV and *Mo*-IDV lambs, we evaluated titers by HI assay (Figure 4A). Lambs showed seropositivity beginning 14 days post-IDV, with all but 2 sal-IDV (3525 and 4016) and all but 2 *Mo*-IDV (3318 and 3811) lambs reaching the defined seropositivity threshold (geometric mean titer (GMT) ≥ 10; sal-IDV GMT = 21.5; *Mo*-IDV GMT = 17.2). Interestingly, overall titers were diminished at the experimental endpoint (22–23 days post-IDV; sal-IDV GMT = 14.1; *Mo*-IDV GMT = 11.2), when all but 2 sal-IDV (3525 and 3418) and all but 3 *Mo*-IDV (3713, 3811, and 4527) lambs remained above the seropositivity threshold. No IDV-neutralizing antibody response was detected in one IDV-positive wether from the *Mo*-IDV group (3811; HI ≤ 5). This lamb shed only low levels of IDV at 6, 8, and 10 DP-IDV (6 DP-IDV: 9.0 ± 17.0 PFU/mL; 8 DP-IDV: 99.0 ± 64.1 PFU/mL; 10 DP-IDV: 86.8 ± 65.2 PFU/mL) and never shed *M. ovipneumoniae,* despite having been inoculated. Conversely, both lambs with subdetectable IDV shedding (sal-IDV: 4016; *Mo*-IDV: 4527) showed a measurable neutralizing antibody response at either 14 days post-IDV (4527; GMT = 16.8) or 22 days post-IDV (4016; GMT = 7.1). Interestingly, *Mo*-IDV lamb 4527 exhibited the lowest shedding of any lamb with detectable *M. ovipneumoniae* and no detectable IDV, but still produced IDV-neutralizing antibodies. Notably, these data support serosurveillance data which suggests that in natural IDV infections, sheep mount a neutralizing antibody response, but that HI titers remain lower than those in some other species such as cattle and swine [33,34,42,47,79].

As with the viral and bacterial shedding data, the HI results were heterogeneous, providing an opportunity for correlative analysis. We found a moderate positive correlation between IDV shedding at 3 days post-IDV and neutralizing antibody response at the endpoint of the study (22 or 23 days post-IDV) (*r_s_* = 0.671, *p* = 0.012). Interestingly, this effect was strongly driven by the *Mo*-IDV lambs (*r_s_* = 0.954, *p* = 0.031) and was not observed within the sal-IDV group (*r_s_* = 0.031, *p* = 0.967) (Figure 4B). Moreover, we also found a strong positive correlation between phase 1 *M. ovipneumoniae* burden and endpoint IDV neutralizing antibody titers (*r_s_* = 0.817, *p* = 0.037) (Figure 4C). Taken together, these correlative data imply a connection between recent *M. ovipneumoniae* burden, early IDV infection, and neutralizing antibody response. Moreover, differential responses between *Mo*-IDV versus sal-IDV lambs suggest that while subtle, recent *M. ovipneumoniae* infection does in some way modify the immunological response to IDV in SPF lambs.

## 4. Discussion

IDV is a recently discovered pathogen, and so far, its pathogenesis has only been studied in a small subset of its natural hosts [33,47,53,58,64,74,80]. The primary goal of this study was to characterize IDV infection in immunologically naïve domestic lambs, which to our knowledge, has not been studied previously. Because lambs are commonly transiently colonized by *M. ovipneumoniae* early in life (2–12 months), we also sought to establish whether IDV progression is altered in the context of a recent or ongoing *M. ovipneumoniae* infection [81]. Our experimental design also allowed us to compare our current findings regarding *M. ovipneumoniae* infection to those we obtained with the previous year’s flock [11]. Finally, we found variable shedding, acute phase responses, and neutralizing antibody titers from lamb to lamb, which we leveraged to identify correlations between recent *M. ovipneumoniae* burden, early-infection IDV replication, and neutralizing antibody response (Figure 2C,D and Figure 4B,C).

In phase 1 of this study, lambs were infected intranasally with a cultured *M. ovipneumoniae* isolate (NW-4) or mock-infected with phosphate-buffered saline, whereas in our previous study, lambs were infected using fresh nasal washes from confirmed *M. ovipneumoniae*-positive sheep [11]. While all nasal wash-inoculated lambs in our previous study sustained a detectable *M. ovipneumoniae* infection for 12 weeks, only 5 of 7 isolate-inoculated lambs in the current study ever detectably shed *M. ovipneumoniae*. Moreover, 6 of 7 lambs in the current study apparently cleared the infection by week 5 (Figure 1A). Importantly, lambs in neither study showed overt clinical signs in response to *M. ovipneumoniae* infection [11]. While our inoculum was a higher dose than that which was previously used by others [25,65,71,72,82,83], it is well established that individual *M. ovipneumoniae* strains vary widely in virulence and that *M. ovipneumoniae* is readily culture-attenuated, both of which could have contributed to our comparatively early *M. ovipneumoniae* clearance and complete lack of respiratory symptoms [28,66,81,84].

In phase 2, all lambs were inoculated with IDV. Similar to *M. ovipneumoniae*, IDV shedding was variable, and 2 lambs failed to detectibly shed virus at any point. Our shedding data show that in sheep, IDV maintains a replication pattern similar to IDVs in other species, inducing an acute infection which persists for less than 2 weeks (Figure 2B and Supplemental Figure S1A,B) [33,47,51,52,53,61]. Moreover, this experiment demonstrated that IDV infection can remain subclinical in lambs, even after recent or ongoing *M. ovipneumoniae* infection, and that virus readily sheds during asymptomatic infection (Figure 2B and Supplemental Figure S1A,B). Although others have observed rectal shedding and viremia in cattle and swine, we found no evidence of either at the chosen timepoints (rectal sample: day 7 post-IDV; blood samples: day 3 post-IDV) [45,47,58]. To date, there are no reported IDV isolates from small ruminant respiratory samples, although there is one report of IDV recovered from a rectal swab from a goat that was critically ill with severe diarrhea [45]. This goat isolate belongs to the same phylogenetic lineage as the IDV used in this study (D/OK) and is closely related to both bovine and swine IDVs, suggesting that some IDVs may contribute to illness (albeit gastric) in sheep under certain circumstances [45]. A more thorough investigation of possible gastrointestinal transmission of IDVs should be pursued in the future to gain insight into this possible mechanism of transmission.

IDV is often asymptomatic in cattle but may predispose its hosts to secondary infections by facultative pathogens, including *Mycoplasma* spp. This effect was demonstrated in a study by Lion and colleagues which found that in immunologically naïve calves, simultaneous IDV-*Mycoplasma bovis* (*M. bovis*) infection increased early-infection *M. bovis* nasal shedding and promoted *M. bovis* colonization in the lower respiratory tract compared to calves that were not coinfected with IDV [64]. Moreover, Nissly and colleagues found that among IDV-positive cattle, those coinfected by additional respiratory pathogens had a higher IDV burden than those solely infected with IDV [63]. While etiology remains unclear, these data suggest that IDV may act synergistically with other respiratory pathogens. In this experiment, *M. ovipneumoniae* was introduced first and had cleared for most lambs by the time IDV was introduced, so for most lambs, any impact of *M. ovipneumoniae* would be due to recent rather than ongoing infection, and therefore, true synergism would not be possible.

Accordingly, in phase 2, we found no difference in clinical symptoms or overall viral shedding between groups. Despite this, we found several indicators that recent *M. ovipneumoniae* infection impacted IDV pathogenesis. For instance, during IDV infection *Mo*-IDV lambs had significantly elevated rectal temperatures compared to the sal-IDV (Figure 1C). While these temperatures always remained subclinical, elevated body temperature is a hallmark response to influenza infection in many species. Interestingly, in our study, *Mo*-IDV group showed a strong positive correlation between total phase 1 *M. ovipneumoniae* burden and early-infection IDV shedding (3 days post-IDV) (Figure 2D). While these data are correlative, one possible explanation is that recent *M. ovipneumoniae* infection expedites the establishment of a subsequent IDV infection. In an immunocompromised host, this might result in a more severe infection, but if the immune system is functioning optimally, as viral burden rises, there is a proinflammatory response which in turn contains the infection to result in asymptomatic carriage, as we observed in these lambs. During an inflammatory response, serum protein levels rise as acute phase proteins are produced. Consistent with our observation of elevated body temperature in *Mo*-IDV lambs during viral infection, we found that *Mo*-IDV total serum protein level was elevated at 14 days post-IDV as compared to 1 day before IDV (Figure 3B). Conversely, serum protein levels in sal-IDV lambs fell for all but one animal, suggesting that recent *M. ovipneumoniae* infection promoted inflammation during IDV infection, whereas IDV alone did not. Collectively, these data suggest the presence of a subclinical immune response to IDV which is enhanced by recent or ongoing *M. ovipneumoniae* infection.

While an innate immunity-driven inflammatory response may partially mitigate an infection, neutralizing antibodies are typically required for complete clearance of a pathogen. Interestingly, hemagglutinin inhibition assays on serum from naturally infected sheep tend to show lower neutralizing antibody titers than those from naturally infected cattle. In a U.S. retrospective study, Quast et al. reported that more than half of seropositive sheep (defined as HI ≥ 1:40) had titers of 1:40, with a maximum documented titer of 1:320 [42]. This is not specific to the United States; in France, no reported titers have exceeded 1:160, and in West Africa, the maximum reported titer to date is 1:80 [38,39,44]. By comparison, HI results in 2400 randomly sampled Irish cattle reported only 7% of seropositive cattle to have titers as low as 1:40, with over 15% of titers ≥ 1:640–1:10,240 [43]. A recent study established that a 1:10 HI cutoff had equivalent specificity and sensitivity to viral microneutralization-based IDV diagnostics, and it may therefore be appropriate to consider whether a lower seropositivity cutoff value should be employed for reporting purposes [85]. Herein we used a cutoff value of GMT ≥ 1:10, i.e., excluding samples for which any replicate is well agglutinated. Mirroring surveillance data, we observed a maximum GMT of 1:67, with a minority of responses exceeding the traditional influenza seropositivity threshold of ≥ 1:40 (sal-IDV = 3/7, *Mo*-IDV = 2/7). Lambs failed to maintain a GMT ≥ 1:40 to the endpoint of the study (22 or 23 days post-IDV), and only 3/6 sal-IDV and 4/7 *Mo*-IDV maintained GMT ≥ 1:10 through the endpoint. These findings are in contrast to three separate calf studies, in which animals sustained or increased their titers between the same timepoints [53,61,64]. Notably, no lambs in this study seroconverted at 6 days post-IDV, which diverges from the response reported in intranasally-inoculated feral swine and calves [47,53], but was consistent with colostrum-deprived calves as well as calves infected by contact transmission [61].

In the abovementioned recent study by Lion and colleagues, increased disease severity in calves coinfected with *M. bovis* and IDV was associated with overexpression of interferon-γ during coinfection as compared to single infections, which the authors attributed to differences in lymphocytic infiltrate between treatment groups [64]. In our study, neither the quality nor the quantity of cellular lung infiltrates at 23 days post-IDV were affected by prior *M. ovipneumoniae* infection. Interestingly, we also found that *M. ovipneumoniae* infection alone does not induce transcription of inflammatory cytokines in PBMCs (data not shown). Nonetheless, our observation of a positive correlation between *M. ovipneumoniae* burden, early-infection IDV shedding, and IDV-neutralizing antibody response suggest that prior *M. ovipneumoniae* infection could promote anti-IDV inflammation. Thus, future studies will examine the quality of anti-viral immune response to IDV infection in lambs and whether and how this response could be modified by persistence of *M. ovipneumoniae*.

Overall, we observed low and unstable neutralizing antibody response to IDV, which may not be uncommon to sheep. Despite this, we found a correlation between IDV shedding at 3 days post-IDV with endpoint HI titer (Figure 4B), suggesting that those lambs with higher early-infection viral loads may mount a more robust secondary immune response and the strength of this correlation was almost exclusively driven by the *Mo*-IDV group (*r_s_* = 0.954, *p* = 0.031). This finding is somewhat surprising because *M. ovipneumoniae* has been shown to directly suppress mitogenic expansion of T-cells and B-cells, which could plausibly result in a delayed or impaired antibody response [31,32]. Interestingly, the lamb with the highest overall *M. ovipneumoniae* burden also had the highest endpoint HI titer (lamb 3318; GMT = 33.6), and *M. ovipneumoniae* burden had a strong positive correlation with endpoint titer, suggesting that recent or ongoing *M. ovipneumoniae* infection may not suppress a heterologous antibody response in lambs (Figure 4C). Taken together, our serological data support prior evidence that sheep fail to either mount or maintain a robust neutralizing antibody response against IDV, and the data indicate that this response may also wane more rapidly than with other hosts, but that recent or ongoing *M. ovipneumoniae* infection does not inhibit antibody production or efficacy.

In conclusion, this study confirms that IDV replicates in sheep and elicits a neutralizing antibody response, but IDV does not confer pathology or strong acute-phase responses even in sheep infected with *M. ovipneumoniae*. Our findings corroborate serosurveillance data that indicate that IDV neutralizing antibody titers appear relatively weak in comparison to other species’ responses, suggesting that they were sufficient to clear the pathogen within two weeks of infection [38,39,42,43,44]. We also noted rapid waning of the antibody response, which could impact serosurveillance study outcomes. Although subclinical, we found multiple indicators that recent *M. ovipneumoniae* infection stimulated an immunological response, featuring elevated body temperature and an increase in total serum protein in response to IDV infection only in lambs previously infected with *M. ovipneumoniae*. Moreover, we found that at day 3, post-IDV viral titers and endpoint HI titers correlated to prior *M. ovipneumoniae* burden. We posit that on their own, both *M. ovipneumoniae* and IDV are minimally immunostimulatory in sheep, but that *M. ovipneumoniae* infection may prime the lamb immune system to respond more robustly to secondary infection by IDV. In the context of two subclinical infections, this may not lead to overt respiratory symptoms, but virulence varies widely between *M. ovipneumoniae* strains [66,81,86], and IDV is not genetically stable [74,87], it readily reassorts [88], and its tissue tropism and host range can vary by strain [74,89]. Changes in any of these parameters could dramatically alter outcomes to coinfection of both of these pathogens in sheep. Finally, recent work has demonstrated that interspecies transmission is possible and has suggested a model wherein cattle are the primary host of IDV, but periodic spillover events result in contained outbreaks in other species [74]. Still, it is unclear whether sheep are an intermediate host and whether sheep adapted IDVs can infect other species. Therefore, it is important not only that IDV surveillance continues, but also that a standardized HI titer cutoff is implemented, and a sheep IDV isolate(s) is obtained and sequenced for reference.

## Figures and Tables

**Table 1:** Clinical parameters from each group at indicated timepoints.

	DPI (*M. ovi*)	DPI (IDV)	sal-IDV (SD)	*Mo*-IDV (SD)
**Attitude ^a^**	2	-	0 (0)	0.14 (0.38)
	3	-	0.14 (0.38)	0 (0)
	14	-	0 (0)	0.17 (0.41)
	21	-	0 (0)	0.14 (0.38)
	23	-	0 (0)	0.14 (0.38)
	42	14	0 (0)	0.14 (0.38)
**Body Condition ^b^**	2	-	3.29 (0.27)	3.36 (0.24)
	9	-	3.29 (0.39)	3.29 (0.39)
	16	-	3.21 (0.27)	3.07 (0.35)
	23	-	3.36 (0.38)	3.57 (0.45)
	30	2	3.64 (0.38)	3.5 (0.29)
	37	9	3.86 (0.24)	3.79 (0.39)
	44	16	3.42 (0.2)	3.57 (0.35)
**Clinical Signs ^c^**	2	-	0 (0)	0.14 (0.38)
	3	-	0.14 (0.38)	0 (0)
	23	-	0 (0)	0.14 (0.38)
	25	-	0.14 (0.38)	0 (0)
	27	-	0 (0)	0.14 (0.38)
	28	0	0 (0)	0.14 (0.38)
	28	0	0 (0)	0.14 (0.38)

^a^ **Attitude**. 0: bright, alert, responsive (BAR, ideal state); 1: less BAR; 2: beginning to appear visibly ill; 3: obviously ill but alert and responsive; 4: depressed, unresponsive; 5: appears critically ill. ^b^ **Body condition**. 1: very lean/bony; 2: lean; 3: ideal; 4: fat; 5: obese. ^c^ **Clinical signs**. 0: healthy; 1: mild discomfort and/or minimal nasal/ocular discharge, panting; 2: clear nasal discharge, attitude change, with or without coughing 1–5 times; 3: major attitude change, constant coughing with decreased appetite and/or moderate nasal or ocular discharge; 4: appears very ill, prominent nasal or ocular discharge, still eating; 5: anorexic, lethargic, unresponsive. DPI: days post-infection; ND: not done.

**Table 2:** Total serum protein and acute-phase response. Start denotes 1 day prior to *M. ovipneumoniae* infection. All other days relative to IDV infection. Endpoint denotes 22 days post-IDV (sal-IDV) or 23 days post-IDV (*Mo*-IDV). SAA: serum amyloid A; P: Bonferroni–Dunn adjusted *p*-value. Comparisons evaluated by Student’s *t*-test.

	Timepoint	sal-IDV			*Mo*-IDV			
		Mean	Median	Range	Mean	Median	Range	*p*
**Total** **Serum Protein (g/dL)**	Start	7.66	7.3	6.2–8.8	7.6	7.6	6.5–8.7	>0.999
	Day-1	10.8	11	9.1–11.7	9.6	9.7	9.1–10.1	0.244
	Day 6	10.4	9.9	7.8–13.1	9	9.5	6.8–10	0.236
	Day 14	10.4	10.3	8–13.5	11.6	11.4	10.5–14.2	0.581
	Endpoint	10.5	10.7	8.6–12.2	10.7	10.5	8.8–13	>0.999
**Albumin (g/dL)**	Start	4.2	4.2	3.9–4.4	4.2	4.3	3.3–4.7	>0.999
	Day-1	4.9	4.9	4.6–5.5	4.8	4.8	3.3–5.9	>0.999
	Day 6	4.9	4.9	4.7–5.3	4.6	4.8	3.3–5.5	>0.999
	Day 14	4.4	4.4	3.9–4.6	4.4	4.4	3.6–5.2	>0.999
	Endpoint	4.6	4.6	4.4–4.7	4.2	4.3	3.1–5	0.546
**SAA (ng/mL)**	Day-1	618.5	58.5	3.4–2902.2	499.7	42.4	0–2936.8	>0.999
	Day 6	158.4	30.8	0–838	667.3	231.2	36.2–2793.5	0.587
**L-lactate (mM)**	Day-1	1.9	2	1.4–2.2	1.7	1.8	1.1–2.3	0.495
	Day 6	1.8	1.9	0.3–3	1.9	1.8	0.8–2.8	0.838

## Data Availability

Not applicable.

## Supplementary Materials

The following supporting information can be downloaded at: https://www.mdpi.com/article/10.3390/v14071422/s1, Figure S1: IDV nasal shedding patterns in SPF lambs varies within both experimental groups.
