# Supplementary material for: Pathophysiology of Influenza D Virus Infection in Specific-Pathogen-Free Lambs with or without Prior Mycoplasma ovipneumoniae Exposure
Source: Viruses. 2022 Jun 28;14(7):1422. doi: 10.3390/v14071422 (PMC9321583; doi:10.3390/v14071422)
Supplement: Supplementary file 1 [file viruses-14-01422-s001.zip › viruses-1714225-supplementary.pdf]

viruses

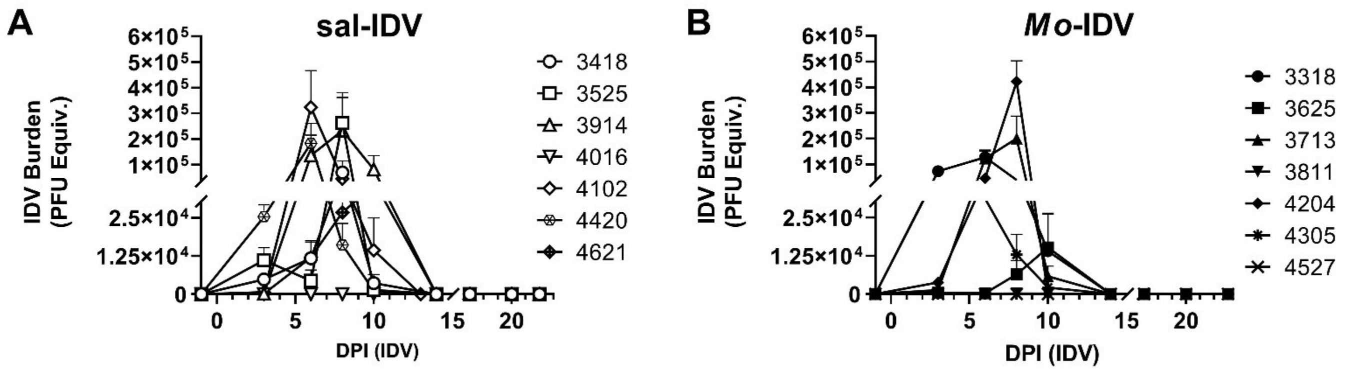

Figure S1. IDV nasal shedding patterns in SPF lambs varies within both experimental groups. IDV burden per nasal swab for individual lambs in (A) the sal-IDV group, or (B) the Mo-IDV group. Data presented as mean of technical replicates ± SD. Enumerated by qRT-PCR using the standard curve method.
